# How Prediction of the Future Affects Encoding of the Present: Cooperation or Competition?

**DOI:** 10.1162/OPMI.a.329

**Published:** 2026-01-15

**Authors:** Brynn E. Sherman

**Affiliations:** Department of Psychology, The Ohio State University, Columbus, OH, USA

**Keywords:** statistical learning, episodic memory, hippocampus, encoding-retrieval trade-offs

## Abstract

Each day brings new experiences and the opportunity to form new episodic memories. However, our everyday experiences are not isolated episodes; rather, there is significant spatial and temporal structure that holds across experiences, allowing us to build up structured knowledge about the world. We can then leverage this known structure to make predictions about how new experiences will unfold. In a recent study in *Open Mind*, Poskanzer, Tarder-Stoll, et al. found that such predictions benefitted episodic memory. Specifically, on trials in which participants were successfully able to make predictions about an upcoming experience, participants were more likely to encode that predictive information into memory. These findings seem to stand in stark contrast to other recent work, which found the opposite of *worse* episodic memory for predictive cues (Sherman et al., 2022; Sherman & Turk-Browne, 2020). How can these discrepant findings be reconciled? Here, I discuss several key task differences that might explain the discrepancy and highlight avenues for future research which might help to theoretically disentangle the contexts under which prediction may impede vs. facilitate episodic encoding.

## INTRODUCTION

Our days are filled with repetitive, statistical structure: Our commutes to work, where we park, and even conversations with colleagues often follow a predictable formula. Yet, layered on top of that structure are unique episodes (e.g., passing by your neighbor decorating for the holidays, finding a bird perched on your car, or being surprised when a colleague unexpectedly brings donuts to a meeting). These facets of experience can interact: If you are in the state of predicting (i.e., thinking about the next turn on your commute), you may be more or less attuned to what’s going on around you, and your memory for the specifics of the commute may be affected accordingly.

How does the predictable structure of experience influence our ability to encode unique details into memory? In other words, when we’re actively leveraging known structure to make predictions about upcoming events, how does that prediction affect our encoding of the current moment? In a recent study, Poskanzer, Tarder-Stoll, et al. (2025; hereafter referred to as PTSA) present findings from three experiments which suggest that prediction of the future can *improve* encoding of the present.

In all three experiments, participants were first exposed to a series of scene exemplars which contained statistical structure in the temporal order of the categories. The statistical structure differed across experiments, but roughly the structure was defined as certain scene subcategories reliably followed by other subcategories. After familiarizing participants to this sequential structure (in an “Initial Structure Learning” Phase), participants underwent a “Simultaneous Prediction and Encoding” Phase in which they were shown novel exemplars from the same scene subcategories and were explicitly asked to make predictions about upcoming image subcategories. This also served as an incidental encoding task, as participants’ memory for these images was subsequently tested in a surprise memory test. The key question was how participants’ memory for these images was influenced by whether they made a successful prediction. They observed trial-by-trial relationships between prediction and memory: successful prediction was associated with better recognition memory for the specific predictive exemplar.

**Figure 1.**
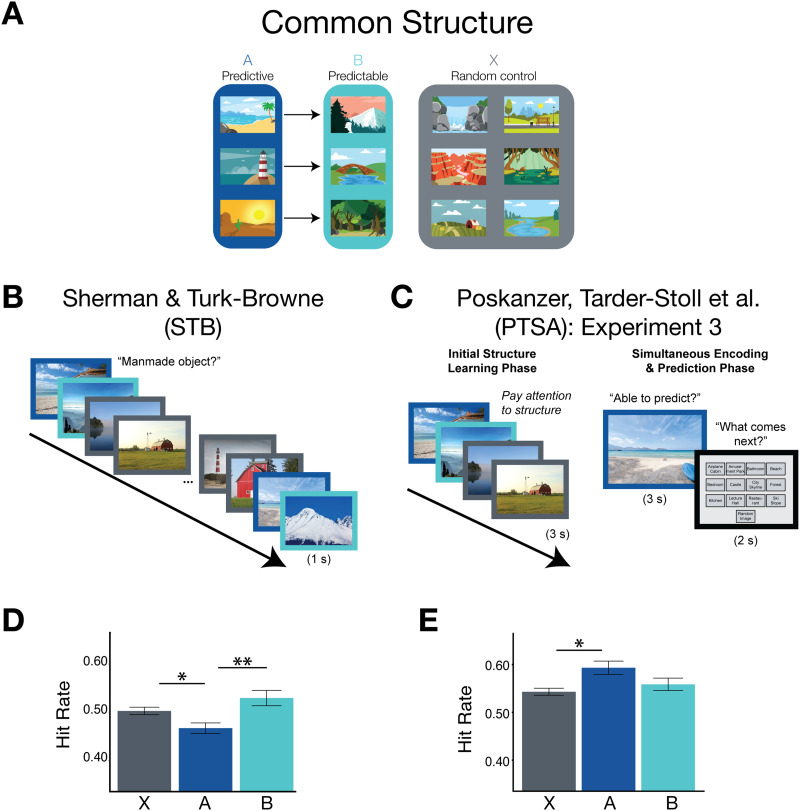
Comparison of paradigms and data from Sherman and Turk-Browne (STB; Experiment 1) and Poskanzer, Tarder-Stoll, et al. (PTSA; Experiment 3). (**A**) In both experiments, participants were exposed to a sequence of scene images, in which certain scene subcategories (predictive A) were reliably followed by others (predictable B). The other scene subcategories were neither predictive nor predictable and thus were randomly inserted throughout the sequence (control X). (**B**) In STB’s study, participants viewed one continuous stream of scene images (following the structure depicted in panel A), during which participants judged whether or not each image contained a manmade object. Each image was presented for 1 second. (**C**) In PTSA’s study, participants first underwent an “Initial Structure Learning Phase” in which they viewed a sequence of images and were instructed that certain scene subcategories followed one another. They subsequently underwent a “Simultaneous Encoding and Prediction Phase” in which each image was presented for 3 seconds and participants were tasked with explicitly reporting whether or not they could predict the category of the upcoming image. They then were shown a screen displaying all 12 scene subcategories (along with a “Random Image” option) and were asked to indicate which of the scene subcategories they predicted would be next. If they had indicated that they couldn’t predict the upcoming image, they were instructed to select the “Random Image” option. (**D**) Data from the surprise memory test in STB’s Experiment 1a. Participants were significantly worse at remembering the predictive A images, relative to both predictable B and control X. (**E**) Data from the surprise memory test in PTSA’s Experiment 3, in which participants were tested on the images encountered in the Simultaneous Encoding and Prediction Phase. Participants were significantly better at remembering the predictive A images, relative to the control X images. Error bars represent ±1 *SEM* within participant.

These data provide provocative evidence that there may be a positive relationship between prediction and encoding. However, as the authors note in their paper, these findings conflict with other work demonstrating a negative relationship between prediction and encoding. Indeed, Sherman and Turk-Browne (2020; hereafter referred to as STB) found that prediction led to *worse* memory. Specifically, in their study, participants were exposed to a sequence of trial-unique scene images, in which certain scene subcategories (*predictive A* categories) reliably preceded others (*predictable B* categories); other scene subcategories (unpaired *X* categories) were included as control items which were neither predictive nor predictable (Figure 1A). Thus, participants could both form a unique episodic memory for each trial-unique scene exemplar, and they could learn to extract the higher-order temporal statistics (i.e., which scene subcategories follow one another). Behaviorally, STB found that memory for the predictive A images was significantly worse, relative to the unpaired control items, suggesting that prediction impeded encoding of the present. Supporting this interpretation, they found that greater evidence of prediction in the hippocampus (as measured via fMRI decoding) was associated with this behavioral trade-off across participants. In subsequent work, they further observed a trial-by-trial negative relationship between memory for predictive items and neural evidence for prediction from intracranial electrodes in visual cortex (Sherman et al., 2022). They took this as evidence for a trade-off between prediction and encoding, such that when the hippocampus can use the current experience to generate a prediction, it is worse at encoding the specifics of the current experience (see also Sherman et al., 2024).

The seemingly opposite pattern of findings from PTSA and STB are puzzling on the surface, especially given the similarity of their paradigms. Indeed, PTSA’s Experiment 3 used the same statistical structure as STB (Figure 1A). That said, there are several differences in the paradigms that—as PTSA note in their General Discussion—may be more than superficial. These differences may provide important theoretical insight into why and when prediction impairs versus improves memory. In this paper, I take a deep dive into the differences between the paradigms in the hopes of (i) resolving some of the discrepancies in past findings and (ii) providing avenues for future research that could lead to a more comprehensive account of the relationship between prediction and memory.

## KEY DIFFERENCES BETWEEN STB AND PTSA’S PARADIGMS

There are three primary differences between STB and PTSA’s paradigms: (1) the pre-exposure to the statistical structure; (2) incidental vs. explicit structure learning and prediction; and (3) differences in encoding time. In the below subsections, I walk through each of these factors and argue that these differences have significant practical and theoretical consequences, which may shed light on the discrepant results between studies.

### Differences in Sequence Exposure: Reducing Temporal Competition Between Episodic Memory and Statistical Learning

In STB’s experiments, participants underwent a single encoding phase. Participants were shown a sequence of scene images and, for each image, asked to make a judgment about whether there was a manmade object in the scene (Figure 1B). This question was chosen to balance the opportunities for episodic encoding (as participants may need to search each image for the manmade object, thus orienting them to the episodic details) and statistical learning (as over time, participants can learn which scene subcategories reliably followed one another). Statistical learning, in turn, can enable prediction, as once the statistics are known, participants can leverage the learned sequential structure to predict the upcoming category (leading to a facilitated response in judging whether the scene contained a manmade object). The experiments’ purpose was thus to see whether either statistical learning-based prediction or episodic encoding would be naturally favored, given the opportunity to use either approach.

PTSA, on the other hand, separated encoding into two phases. Participants first underwent an Initial Structure Learning Phase, in which they were pre-exposed to sequences that contained the temporal statistics and were explicitly tasked with uncovering the hidden temporal structure (Figure 1C, left). This was then followed by a Simultaneous Encoding and Prediction Phase, in which participants made predictions while incidentally encoding the images (Figure 1C, right).

Thus, whereas in STB’s study, participants were learning the temporal statistics at the same time that they were encoding, PTSA’s design front-loaded the learning of the temporal statistics. This difference is important insofar as STB’s effects may critically depend on whether statistical learning and episodic encoding are in temporal competition with one another. For example, in interpreting their evidence for trade-offs, STB argue when the statistics of the environment are noisy (i.e., during learning) it may be more adaptive to make a (testable) prediction about what might be coming next, rather than using those resources to precisely encode the current experience (which is largely redundant with what already exists in memory). However, if learning is complete by the time of encoding (as is perhaps true in PTSA’s study), then resources instead might be re-allocated to encoding the idiosyncratic details of a given experience. That is, to the extent to that prediction and memory trade-off with one another in time (see further discussion of this in the subsection Differences in Encoding Time: Temporal Dynamics of Encoding/Retrieval Trade-Offs below), then as predictions become better learned and more efficient, more time can be devoted to episodic encoding. This account would predict a shift in the relationship between prediction and encoding over an extended period of learning: (Relatively) early on, there may be evidence for a trade-off, but as the temporal statistics becomes over-learned, there may be a facilitation. Because STB only probe the relatively early stages of learning (with relatively few exposures, compared to prior work using a similar paradigm; Brady & Oliva, 2008) and PTSA only test memory for the images that were presented after extensive explicit structure learning, this possibility cannot be assessed in either current dataset. Future work directly manipulating the amount of pre-exposure would help to disentangle these possibilities.

### Differences in Explicit Orientation to Structure: Consequences for Prediction Strength

During the Initial Structure Learning Phase, in which participants were pre-exposed to the sequential structure, PTSA explicitly tasked participants with uncovering the hidden structure. This presents another difference relative to STB, where the learning of structure was fully incidental. Whether and how the explicitness of learning might affect the nature of the learning process—as well as subsequent memory outcomes—is an open question. For example, it is possible that more explicit learning of structure in PTSA’s studies engages different processes than traditional, implicit “statistical learning” (Batterink et al., 2015). That said, there is mixed evidence regarding the extent to which explicit knowledge affects statistical learning, with some studies finding no differences in statistical learning under explicit vs. implicit conditions (e.g., Arciuli et al., 2014; see further discussion in Siegelman & Frost, 2015).

Nevertheless, the choice to explicitly orient participants to the structure was consequential insofar as it enabled another key design difference. Specifically, in PTSA’s experiments, prediction was *explicit*: On every trial of the Simultaneous Encoding and Prediction Phase, participants were tasked with predicting the upcoming scene subcategory (Figure 1C, right). This contrasts with STB’s experiments, in which prediction was *incidental* and occurred as a consequence of ongoing statistical learning.

Together, the explicit task of prediction, along with the extensive pre-exposure to the sequential structure and the explicit orientation to the structure during the Initial Structure Learning Phase, meant that participants in PTSA’s study were likely making ‘stronger’, more certain predictions than participants in STB’s study. How might the strength of prediction interact with memory encoding? On the one hand, the explicit task of prediction might have led to even greater competition between prediction and memory (i.e., a stronger version of what STB found): If prediction and memory trade-off, then one might expect that stronger predictions would be associated with worse encoding. Relatedly, because participants in PTSA’s study were tasked with explicit prediction (while encoding was incidental), one might expect that participants would simply follow the instruction to predict and, consequently, not encode the details, leading to an apparent trade-off. On the other hand, however, it is possible that as predictions become stronger and more certain, there is not as much need for competition: Prediction can occur more easily and efficiently, and resources can instead be dedicated to encoding. PTSA’s data perhaps provide some evidence for the latter possibility: In their Experiment 1, participants were tasked with predicting images that would occur 1–4 steps in the future. The effects scaled with prediction distance, such that closer predictions were faster, more accurate, and associated with better memory, suggesting that prediction strength indeed might affect memory in a graded fashion.

That said, it is unclear how exactly the explicit orientation to structure and/or prediction might be affecting prediction strength. It is worth noting, for example, that prediction accuracy in PTSA’s experiments ranged from an average of 60–73%. Although this is not ceiling performance, it is unclear what level of accuracy to expect if structure learning and/or prediction were not explicit (as STB did not behaviorally measure prediction accuracy). Thus, future work manipulating explicit knowledge of the structure and/or whether predictions are made explicitly vs. implicitly will be important for understanding the relationship between prediction strength and memory outcomes.

### Differences in Encoding Time: Temporal Dynamics of Encoding/Retrieval Trade-Offs

The choice to explicitly test participants’ predictions on each trial required another key change to the task: Participants had to be given considerably more time to view and respond to each image. Whereas STB presented each image for 1s (during which participants presumably encoded each image and made their prediction), PTSA presented their images 3–4 times longer (depending on the experiment).

The timing matters insofar as both PTSA and STB suggest that their findings may arise from the need to balance between two mutually exclusive processing states in the hippocampus. Specifically, an influential model of the hippocampus (e.g., Hasselmo, 2005; Hasselmo et al., 2002) proposes that the hippocampus toggles between an *encoding* state (supporting encoding of the external environment) and a *retrieval* state (supporting retrieval of past memories). Behavioral work in humans supports the notion that the two states are mutually exclusive, such that encoding and retrieval cannot co-occur in time (Duncan et al., 2012; Patil & Duncan, 2018). Relating these processing states to their data, STB argue that when participants are engaged in prediction, they are in a ‘retrieval state’ in which they are drawing from past experiences to generate a prediction. Being in such a ‘retrieval state’ thus renders them unable to encode the details (i.e., be in a simultaneous ‘encoding state’). PTSA, on the other hand, posit that once prediction is complete (i.e., successful), participants can efficiently switch back to an encoding state, driving a positive relationship between prediction success and encoding success.

The idea that encoding and retrieval states might dynamically toggle based on the outcome of one state is an intriguing possibility that will need to be explored in further work. However, if this mechanism is at play, then why would STB have ever observed a trade-off? The difference in encoding time across the studies may play a critical role in determining whether encoding and prediction interfere with or facilitate one another. It could be the case that when the system is under strain (because there is limited time to both encode and predict, as in STB), a trade-off will emerge. But, if there is sufficient time to switch between encoding and retrieval (as in PTSA; enabling resources to be dedicated to both processes), then the two processes could co-exist or even facilitate each other.

This dissociation is perhaps consistent with PTSA’s Experiment 1, in which participants were tasked with making predictions that were 1–4 steps in the future. They found that as prediction distance increased, memory encoding decreased (controlling for prediction success). This supports the notion that time may arbitrate between prediction and encoding: When predictions are farther in the future and thus more effortful (supported empirically by longer response times), then there is less time to switch back into a successful encoding state, leading to a trade-off. The time-dependency of trade-offs is also supported by prior behavioral work demonstrating competition between encoding and retrieval states for short (.5–1 s) but not long (1.5+ s) inter-stimulus intervals (Duncan et al., 2012; Patil & Duncan, 2018). Future work using time-resolved methods to track encoding and retrieval states (e.g., Long & Kuhl, 2019) may be useful in fortifying the links between encoding/retrieval dynamics and behavior.

## OPEN QUESTIONS AND BROADER IMPLICATIONS

In the above section, I outlined some key design differences between PTSA and STB that may explain the divergent results. Although these factors bring us a step closer to understanding why prediction might both facilitate and impair memory, there are still several open questions about how precisely prediction and memory interact. Here, I focus on three broad questions: (1) Whether the effects of prediction on memory may depend on the accuracy of the prediction; (2) What mechanisms within the hippocampus might mediate the relationship between prediction and encoding; and (3) How to unify the inter-related concepts of retrieval, prediction, and statistical learning.

### Act of Prediction or the Consequences of Prediction?

Fundamentally, STB claim that the process of generating a prediction is what leads to impaired encoding of the present—regardless of whether that prediction is successful or not. PTSA, on the other hand, make a specific claim about *successful* prediction. They propose that when prediction is successful, the retrieval state can end, leading to more efficient switching back to an encoding state, promoting enhanced encoding. However, at the time of prediction, how could the brain know whether a prediction is successful or not? Without explicit feedback, the mind would only know whether it’s predicting or not, and as long as it’s in a retrieval state (i.e., the act of predicting), then it should not be able to be in an encoding state. One might thus expect the results to track with prediction speed or prediction confidence, rather than prediction accuracy. In other words, the switch back to an encoding state may occur whenever the act of predicting is complete, rather than being modulated by whether the prediction is accurate.

The idea that something akin to prediction completion, rather than accuracy, may matter is perhaps consistent with PTSA’s framing of their findings: They argue that the switch between a retrieval and encoding state may be governed by whether or not participants meet the “retrieval goal” of prediction. However, because their primary outcome measure is prediction *accuracy*, it is difficult to disentangle whether their results truly track with accuracy or “prediction completion.”

This distinction is important, as it has consequences for the potential mechanism by which prediction and encoding interact. If prediction accuracy, rather than completion, tracks memory, this may suggest that the facilitation between prediction and encoding is not something which occurs online (due to shifting encoding and retrieval states), but rather is something which occurs retroactively, at the time of prediction confirmation. One possibility, for example, is that when a prediction is confirmed, participants reactivate the predictive (A) item while viewing the predicted (B) item as a means of reinforcing the association. When a prediction is violated, on the other hand, participants may try to actively suppress the association between the two items, leading to a retroactive forgetting of the predictive one (perhaps via a nonmonotonic plasticity mechanism; Ritvo et al., 2019). Such an effect could explain how prediction accuracy could track memory performance, even without an online interaction between the two. The critical point is that it is important to disentangle prediction itself from the *consequences* of prediction. There is a large body of work on the effects of predictions vs. prediction errors on memory for *predictable* information (e.g., Bein et al., 2023; Greve et al., 2019; Huang et al., 2025). However, such work does not examine the consequences of prediction/prediction error for memory of the *predictive* cue, as in the PTSA and STB studies.

That said, there is at least some evidence in PTSA’s data to argue against this “offline” interpretation. Specifically, in Experiments 1–2, participants received no “feedback” about whether their predictions were correct: Images in the Simultaneous Encoding and Prediction Phase were presented in a random order, such that, although participants made a prediction on every trial, the following trial was not diagnostic of whether their prediction was correct. However, in Experiment 3, the learned temporal structure was preserved during the Simultaneous Encoding and Prediction Phase, such that participants received implicit feedback about whether their prediction were correct. They found the same pattern of results across both manipulations, suggesting that prediction confirmation (i.e., learning whether the prediction was indeed accurate) may not play a significant modulating role, at least in their current design. It is worth noting, however, that prediction accuracy may be confounded with “time to predict” in their study: Accurate predictions tended to be faster, making it difficult to disentangle the two. However, there may be circumstances in which accuracy and confidence diverge (e.g., with limited data, participants may pick up on spurious temporal relationships, leading to confident, but inaccurate predictions), and testing such cases would be crucial for addressing whether the interaction between prediction and memory is governed by accurate vs. complete predictions.

Time-resolved neural measures might also provide insight into whether prediction and memory interact online (as a function of the prediction itself) vs. offline (as a function of the prediction outcome). For example, Sherman et al. (2022) used intracranial recordings from epilepsy patients to track evidence for prediction and relate to memory outcomes. They found that evidence for prediction specifically during the inter-stimulus-interval period after the predictive (A) but before the predictable (B) items related to worse memory for the predictive item. A similar time-resolved approach may be fruitful in understanding how the processing of the prediction outcome is related to subsequent memory performance. It is possible, for instance, that there are dissociable neural signals during the predictive vs. predictable cue, which combined give rise to improved (or impaired) memory for predictive items.

### Hippocampal Mechanisms Underlying the Relationship Between Prediction and Memory?

PTSA took inspiration from and frame their findings in light of encoding and retrieval modes in the hippocampus (Hasselmo, 2005). Although STB invoke the idea of encoding-retrieval trade-offs in interpreting their results and cite that as a potential mechanism, their study was primarily inspired by the C-HORSE model (Schapiro et al., 2017; Sučević & Schapiro, 2023; Zhou et al., 2023), which proposes that separate subcircuits within the hippocampus can support episodic memory and statistical learning (i.e., the integration across related memories, which enables prediction). Whereas competition in the Hasselmo model arises from mutually exclusive encoding and retrieval modes, a conflict in C-HORSE could theoretically arise because subfield CA1 is part of both hippocampal circuits. This means that CA1 could be a computational bottleneck, determining whether episodically or statistically encoded information will be represented. Although this possibility has not been fully explored within C-HORSE, there is some evidence for trade-offs across the two pathways (Sučević & Schapiro, 2023). Thus, STB hypothesized that when faced with the simultaneous goals of prediction (from statistical learning) and episodic encoding, competition between hippocampal circuits leads to a trade-off.

**Figure 2.**
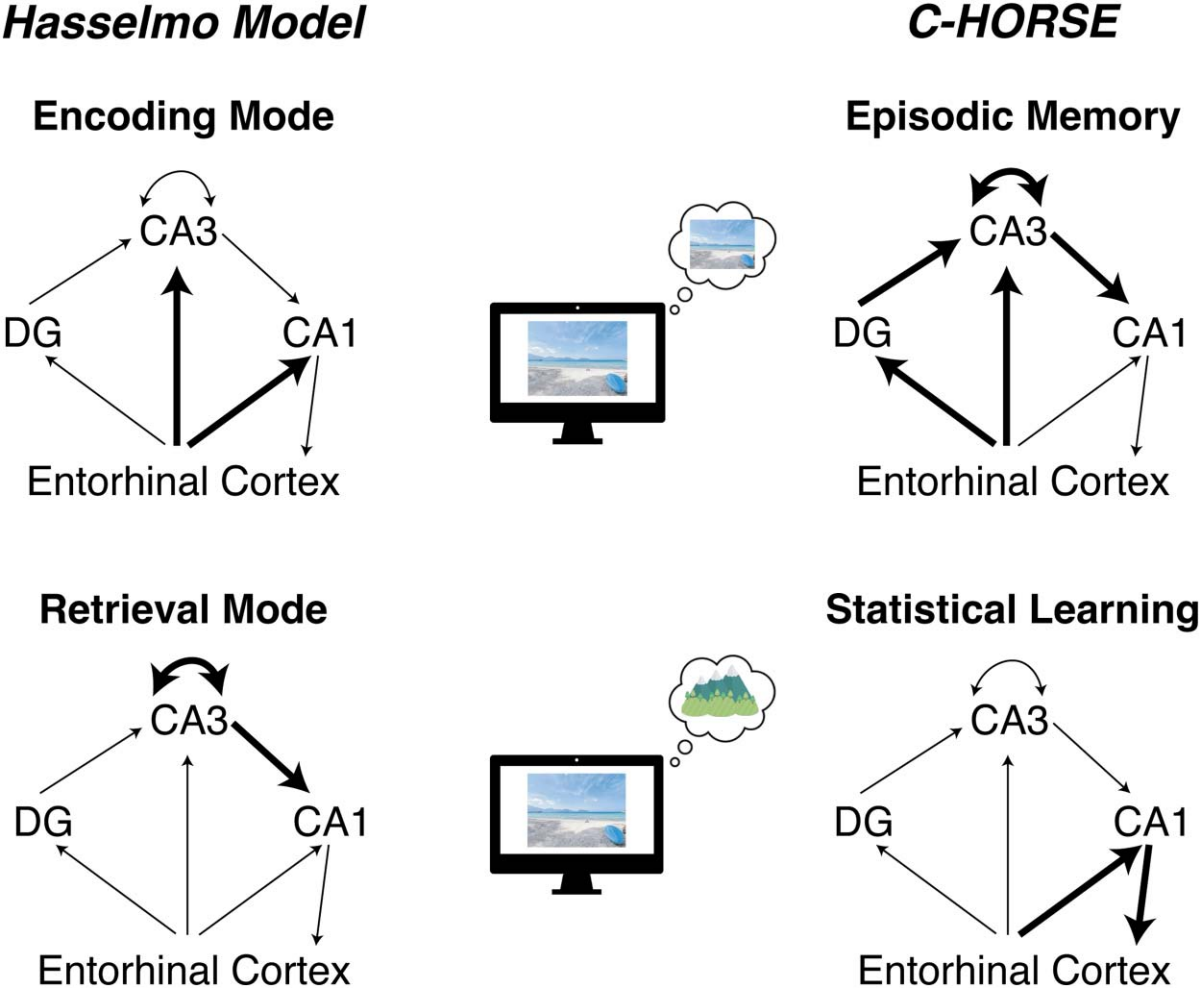
Comparison of Hasselmo model and C-HORSE. In the Hasselmo model, the ‘encoding mode’ (top left) is marked by a relatively greater influence of Entorhinal Cortex (EC) inputs on the hippocampus. In contrast, in C-HORSE, episodic memory (top right), depends on the pathway from EC ➔ CA3/DG ➔ CA1. Despite these (partially) opposing pathways, both support the pattern-separated behavior required for encoding the episodic details of the current experience (top middle). In Hasselmo’s ‘retrieval mode’ (bottom left), there is a relative strengthening of recurrent CA3 connections and CA3 ➔ CA1 connections, which supports an internal focus on retrieving prior memories (bottom middle). In C-HORSE, statistical learning (which enables prediction, presumably relying on retrieval), depends on the EC ➔ CA1 pathway.

Both models are instantiated in the same underlying hippocampal circuitry (see Figure 2). In fact, the learning mechanisms implemented in C-HORSE were inspired by the encoding-retrieval dynamics of the Hasselmo model (Ketz et al., 2013). In spite of this, the mapping of the two models to one another is not straightforward. In the Hasselmo model, the pathway from entorhinal cortex (EC) ➔ CA1 primarily plays a role in *encoding*. In C-HORSE, on the other hand, EC ➔ CA1 supports the specific process of *statistical learning*, which may be computationally opposing to “encoding”. Although it is outside of the scope of this paper to reconcile the discrepancies between the two models, it is worth noting that the models make distinct predictions about the mapping of different kinds of memory-based behavior onto different hippocampal subcircuits.

Ultimately, the key question is whether either or both of these models can account for the bi-directional effects of prediction on memory observed in PTSA and STB. One possibility, for instance, is that the Hasselmo model could account for both effects by manipulating the timing of the switch between retrieval and encoding modes. Under current instantiations of the model, the encoding state occurs at the peak of the CA1 theta cycle, and the retrieval state occurs at the trough, such that encoding and retrieval oscillate at the frequency of theta. Perhaps that timing could produce the trade-offs that STB observed. PTSA’s data could then perhaps be accommodated by the model by making the switch between encoding and retrieval governed not by theta but by the act of prediction/retrieval. Such a finding would suggest that the timing of encoding-retrieval dynamics could explain the discrepancies between PTSA and STB, and would generate predictions about how the timing of inputs from EC vs. CA3 onto CA1 might explain both positive and negative relationship between prediction and memory.

Another possibility is that C-HORSE might be able to account for both effects: More recent instantiations of C-HORSE (Zhou et al., 2023) provide mechanisms by which the strength of the pathways could be modulated. For example, perhaps when the monosynaptic pathway, the pathway from EC ➔ CA1 which supports statistical learning, is more dominant (thus exerting a greater influence on the CA1 bottleneck), then the model would produce trade-offs akin to STB’s results. But if the trisynaptic pathway, the pathway from EC ➔ CA3/DG ➔ CA1 which supports episodic memory, is more dominant, then memory encoding might be enhanced, per PTSA’s data (though it is unclear how this would lead to a facilitation across the two pathways/processes). It is also possible that changing the relative influences of the pathways on CA1 as a function of time might be useful in understanding whether C-HORSE can produce both trade-offs and facilitations. Such patterns would then raise questions about what factors might be controlling the influence of the two pathways on CA1 representations.

Grounding the behavior in computational models can also allow for tighter predictions about the distinct roles of hippocampal subfields in supporting different types of memory behavior (which could subsequently be empirically tested). Thus, understanding the relationship between behavior and hippocampal mechanisms holds promise not only for delineating when prediction facilitates vs. interferes with memory encoding, but also for deepening our understanding of how hippocampal circuits functionally support memory formation.^1^

### Statistical Learning, Prediction, and Retrieval: One and the Same?

A close scrutiny of the aforementioned models also reveals important gaps in our understanding of the relationship between the key memory constructs studied by STB and PTSA. For example, in interpreting their results with respect to the Hasselmo model, STB and PTSA assume that predictions (which were presumably acquired via statistical learning) are a type of ‘retrieval’, as the Hasselmo model has no distinction between ‘prediction’ and ‘retrieval’. Is this a valid assumption?

The Hasselmo model also does not distinguish between “episodic memory” and “statistical learning”. This raises an important issue: Are predictions from statistical learning the same thing as predictions from episodic memory? Possibly not: Whereas a prediction from episodic memory may be more phenomenologically akin to a direct ‘retrieval’, predictions from statistical learning may be noisier and/or less precise, as they reflect abstraction across multiple episodes. For example, if you’re trying to decide how early to arrive for a flight at your local airport over a holiday weekend, you might rely heavily on your most recent holiday travel experience at that specific airport (i.e., by retrieving your episodic memory for that experience and applying it to make a prediction about what the airport may be like this time). On the other hand, if you’re traveling through an airport that you’ve never been to before, you might instead rely on your aggregated experiences across several different trips, leading to a more general prediction that isn’t necessarily grounded in specific, episodic details. Recent work has suggested that the hippocampal mismatch signals specifically respond to episodic, but not semantic, prediction errors (Varga et al., 2025), providing an initial suggestion that different types of prediction may rely on distinct mechanisms. Whether and how one type of prediction may be more akin to a canonical ‘retrieval’ mechanism remains an important open question that bears on whether encoding/retrieval trade-offs of the Hasselmo model can explain the results of STB and/or PTSA.

The question of whether predictions from statistical learning differ from predictions from episodic memory relates to another important question: Is there a difference between the *process of statistical learning* and *making a prediction from statistical learning*? In other words, whereas acquiring statistical regularities may depend on the “statistical learning” pathway of the hippocampus (per C-HORSE), perhaps applying learned regularities (in the service of prediction) is a different computational process with different neural substrates (perhaps supported by a ‘retrieval’ mechanism). Some work points to a common mechanism: In recent modeling work, Singh et al. (2025) found that subfield CA1, which supports statistical learning in C-HORSE, can account for prediction-based memory distortions, suggesting that a single subfield may be responsible both for learning the regularities and supporting prediction-based behavior. Further investigation into whether this is one or two processes will be important for (i) reconciling the Hasselmo and C-HORSE models, and (ii) understanding why the relationship between encoding and prediction may change over the course of learning.

Finally, understanding the nature of the relationship between statistical learning, prediction, and retrieval will require understanding whether predictions in PTSA’s experiments were in fact driven by statistical learning. Given that participants were explicitly tasked with learning the structure, it is possible that they engaged different mechanisms, such that their learning and predictions were more episodic in nature. In other words, it is possible that STB’s participants engaged in prediction from statistical learning whereas PTSA’s participants engaged in prediction from episodic memory. However, regardless of whether predictions arose from statistical learning vs. episodic memory, it is worth noting that, given the nature of the task, the predictions that participants make were inherently probabilistic in nature. Because each individual scene exemplar is novel, predictions—whether through statistical learning or through episodically retrieving a past exemplar—can only go so far: If a participant predicts that a beach image will appear next, they may be predicting blue sky, sand, and ocean, but they cannot predict all of the idiosyncrasies of the image-to-be-shown. However, because both PTSA and STB only probe recognition memory for whole images, they are not sensitive to whether the *specifics* of the prediction matter. In other words, it is possible that prediction and memory interact in a feature-specific way: Perhaps prediction specifically impairs or enhances memory for the features of an event that were consistent (or inconsistent) with the prediction. Addressing this question will be important for understanding the precise relationship between prediction and memory encoding.

## CONCLUSION

Taken together, the studies of PTSA and STB raise an intriguing question: How and why can prediction have opposing influences on episodic memory encoding? Unraveling this mystery has consequences far beyond understanding the methodological differences of the studies. Rather, understanding the circumstances under which prediction helps vs. hinders new memory formation has deep implications for understanding how our memory systems interact and give rise to adaptive, future-oriented behavior.

## ACKNOWLEDGMENTS

For valuable feedback on the manuscript, I am grateful to Mariam Aly, Dhairyya Singh, Hannah Tarder-Stoll, Nick Turk-Browne, Tristan Yates, and Sami Yousif. For assistance in analyzing PTSA’s data, I thank Jie (Skye) Zheng.

## Note

^1^ I have focused on the role of the hippocampus in arbitrating between these learning and memory processes, given the prominence of these models and their influence in the framing of both PTSA and STB’s studies. However, there are likely many other brain regions at play in arbitrating between these learning processes. For example, large parts of sensory cortex are involved in prediction and statistical learning (Sherman et al., 2022, 2023), and the hippocampus perhaps works in concert with these regions to support statistical learning (Zhou & Turk-Browne, 2025). Given that memory encoding also involves cortical representations (e.g., Lee et al., 2019), it is possible that some interaction between prediction and memory may occur within cortex. Additionally, there is likely involvement of top-down control regions (perhaps medial prefrontal cortex; see Sherman et al., 2024) onto the hippocampus, which may govern the way the hippocampus encodes statistical vs. episodic information.
